# Cluster Flow: A user-friendly bioinformatics workflow tool

**DOI:** 10.12688/f1000research.10335.2

**Published:** 2017-05-02

**Authors:** Philip Ewels, Felix Krueger, Max Käller, Simon Andrews

**Affiliations:** 1 Department of Biochemistry and Biophysics, Science for Life Laboratory, Stockholm University, Stockholm, Sweden; 2 Bioinformatics Group, The Babraham Institute, Cambridge, UK; 3 Science for Life Laboratory, School of Biotechnology, Division of Gene Technology, Royal Institute of Technology, Stockholm, Sweden

**Keywords:** Workflow, Pipeline, Data analysis, Parallel computing, Next-generation sequencing, Bioinformatics

## Abstract

Pipeline tools are becoming increasingly important within the field of bioinformatics. Using a pipeline manager to manage and run workflows comprised of multiple tools reduces workload and makes analysis results more reproducible. Existing tools require significant work to install and get running, typically needing pipeline scripts to be written from scratch before running any analysis. We present Cluster Flow, a simple and flexible bioinformatics pipeline tool designed to be quick and easy to install. Cluster Flow comes with 40 modules for common NGS processing steps, ready to work out of the box. Pipelines are assembled using these modules with a simple syntax that can be easily modified as required. Core helper functions automate many common NGS procedures, making running pipelines simple. Cluster Flow is available with an GNU GPLv3 license on GitHub. Documentation, examples and an online demo are available at http://clusterflow.io.

## Introduction

As the field of genomics matures, next-generation sequencing is becoming more and more affordable. Experiments are now frequently run with large numbers of samples with multiple conditions and replicates. The tools used for genomics analysis are increasingly standardised with common procedures for processing sequencing data. It can be inconvenient and error prone to run each step of a workflow or pipeline manually for multiple samples and projects. Workflow managers are able to abstract this process, running multiple bioinformatics tools across many samples in a convenient and reproducible manner.

Numerous workflow managers are available for next-generation sequencing (NGS) data, each varying in its approach and use. Many of the popular tools allow the user to create analysis pipelines using specialised domain specific languages ( *Snakemake* ^1^, *NextFlow* ^2^, *Bpipe* ^3^). Such tools allow users to rewrite existing shell scripts into pipelines and are principally targeted at experienced bioinformaticians with high throughput requirements. They can be used to create highly complex analysis pipelines that make use of concepts, such as divergent and convergent data flow, logic checkpoints and multi-step dependencies. Using such a free-form approach allows great flexibility in workflow design.

Whilst powerful, this flexibility comes at the price of complexity. Setting up new analysis pipelines with these tools can be a huge task that deters many users. Many NGS genomics applications don’t require such advanced features and can instead be run using a simple, mostly linear, file based system. Cluster Flow aims to fill this niche: numerous modules for common NGS bioinformatics tools come packaged with the tool ( Supplementary Table 1), along with ready to run pipelines for standard data types. By using a deliberately restricted data flow pattern, Cluster Flow is able to use a simple pipeline syntax. What it lacks in flexibility it makes up for with ease of use; sensible defaults and numerous helper functions make it simple to get up and running.

Cluster Flow is well suited to those running analysis for low to medium numbers of samples. It provides an easy setup procedure with working pipelines for common data types out of the box, and is great for those who are new to bioinformatics.

## Methods

### Implementation

Cluster Flow is written in Perl and requires little in the way of installation. Files should be downloaded from the web and added to the user’s bash PATH. Command line wizards then help the user to create a configuration file. Cluster Flow requires pipeline software to be installed on the system and directly callable or available as environment modules, which can be loaded automatically as part of the packaged pipelines.

### Operation

Cluster Flow requires a working Perl installation with a few minimal package dependencies, plus a standard bash environment. It has been primarily designed for use within Linux environments. Cluster Flow is compatible with clusters using Sun GRIDEngine, SLURM and LSF job submission software. It can also be run in ’local’ mode, instead submitting background jobs using bash.

Pipelines are launched using the cf Perl script, with input files and other relevant metadata provided as command line options. This script calculates the required jobs and launches jobs accordingly.

### Modules and pipelines

Cluster Flow uses modules for each task within a pipeline. A module is a standalone program that uses a simple API to request resources when Cluster Flow launches. The module then acts as a wrapper for a bioinformatics tool, constructing and executing a suitable command according to the input data and other specified parameters. The online Cluster Flow documentation contains extensive documentation about how to write new modules, making it possible for users to create new modules for missing tools.

Where appropriate, modules can accept param modifiers on the command line or in pipeline scripts that change the way that a module runs. For example, custom trimming options can be supplied to the *Trim Galore!* module to change its behaviour. The parameters accepted by each module are described in the Cluster Flow documentation.

Modules are strung together into pipelines with a very simple pipeline configuration script ( Supplementary Figure 1). Module names are prefixed with a hash symbol ( #), and tab spacing indicates whether modules can be run in parallel or in series. Parameters recognised by modules can be added after the module name or specified on the command line to customise behaviour.

### Genomes

**Figure 1.**
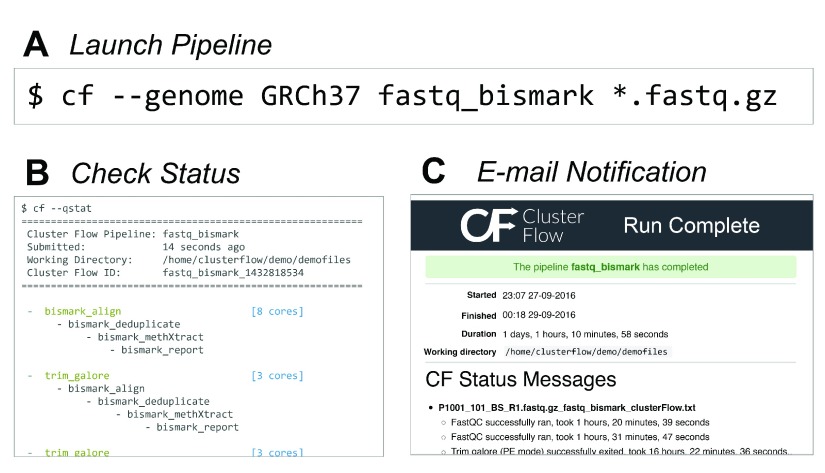
Process for ( **A**) Launching an analysis pipeline, ( **B**) checking its status on the command line and ( **C**) a typical notification e-mail.

Cluster Flow comes with integrated reference genome management. At its core, this is based on a configuration file listing paths to references with an ID and their type. An interactive command line wizard helps with building this file, able to automatically search for common reference types. Once configured, the genome ID can be specified when running Cluster Flow, making multiple reference types available for that assembly. This makes pipelines simple and intuitive to launch ( Figure 1A).

### Pipeline Tracking

Unlike most other pipeline tools, Cluster Flow does not use a running process to monitor pipeline execution. Instead, it uses a file-based approach, appending the outputs of each step to ‘.run‘ files. When running in a cluster environment, cluster jobs are queued using the native dependency management. Cluster Flow can also be run locally, using a bash script in a background job to run modules in series. The current status can be queried using a subcommand, which prints the queued and running steps for each pipeline along with information such as total pipeline duration and the working directory ( Figure 1B).

### Notifications and logging

When pipelines finish, Cluster Flow automatically parses the run log files and builds text and HTML summary reports describing the run. These include key status messages and list all commands executed. Any errors are clearly highlighted both within the text and at the top of the report. This report is then e-mailed to the user for immediate notification about pipeline completion, clearly showing whether the run was successful or not ( Figure 1C).

Cluster Flow modules collect the software version of the tools used when they run. These are standardised, saved to the log files and included in the summary e-mail upon pipeline completion. Cluster Flow logs are recognised by the reporting tool *MultiQC* ^4^ allowing reporting of software versions and pipeline details in MultiQC reports alongside output from the pipeline tools. System information ( PATH, user, loaded environment modules, sysinfo) is also logged to the submission log when a pipeline is started.

### Helper functions

Much of the Cluster Flow functionality is geared towards the end-user, making it easy to launch analyses. It recognises paired-end and single-end input files automatically, grouping accordingly and triggering paired-end specific commands where appropriate. Regular expressions can be saved in the configuration that will automatically merge multiplexed samples before analysis and FastQ files are queried for encoding type before running. If URLs are supplied instead of input files, Cluster Flow will download and run these, enabling public datasets to be obtained and analysed in a single command. Cluster Flow is also compatible with *SRA-explorer* ( https://ewels.github.io/sra-explorer/), which fetches download links for entire SRA projects. Such features can save a lot of time for the user and prevent accidental mistakes when running analyses.

## Use cases

Cluster Flow is designed for use with next-generation sequencing data. Most pipelines take raw sequencing data as input, either in FastQ or SRA format. Outputs vary according to the analysis chosen and can range from aligned reads (eg. BAM files) to quality control outputs to processed data (eg. normalised transcript counts). Tool wrappers are written to be as modular as possible, allowing custom data flows to be created.

The core Cluster Flow program is usually installed centrally on a cluster. This installation can have a central configuration file with common settings and shared reference genome paths. Users can load this through the environment module system and create a personal configuration file using the Cluster Flow command line setup wizard. This saves user-specific details, such as e-mail address and cluster project ID. In this way, users of a shared cluster can be up and running with Cluster Flow in a matter of minutes.

Cloud-computing is becoming an increasingly practical solution to the requirements of high-throughput bioinformatics analyses. Unfortunately, the world of cloud solutions can be confusing to newcomers. We are working with the team behind Alces Flight ( http://alces-flight.com) to provide a quick route to using the Amazon AWS cloud. Alces Flight provides a simple web-based tool for creating elastic compute clusters which come installed with the popular Open Grid Scheduler (SGE). Numerous bioinformatics tools are available as environment modules, compatible with Cluster Flow. We hope that Cluster Flow will soon be available and preconfigured as an such an app, allowing a powerful and simple route to running analyses in the cloud in just a few minutes with only a handful of commands.

Finally, Cluster Flow can also easily be used on single node clusters in *local* mode, as a quick route to running common pipelines. This is ideal for testing, though as there is no proper resource management it is not recommended for use with large analyses.

## Conclusions

We describe Cluster Flow, a simple and lightweight workflow manager that is quick and easy to get to grips with. It is designed to be as simple as possible to use - as such, it lacks some features of other tools such as the ability to resume partially completed pipelines and the generation of directed acyclic graphs. However, this simplicity allows for easy installation and usage. Packaged modules and pipelines for common bioinformatics tools mean that users don’t have to start from scratch and can get their first analysis launched within minutes. It is best suited for small to medium sized research groups who need a quick and easily customisable way to run common analysis workflows, with intuitive features that help bioinformaticians to launch analyses with minimal configuration.

## Software availability

Cluster Flow available from: http://clusterflow.io

Source code available from: https://github.com/ewels/clusterflow

Archived source code as at time of publication: https://doi.org/10.5281/zenodo.57900

License: GNU GPLv3

## Supplementary material

**Typical pipeline script and a list of modules with tool description and URL.** The script shows the analysis pipeline for reduced representation bisulfite sequencing (RRBS) data, from FastQ files to methylation calls with a project summary report. Pipeline steps will run in parallel for each read group for steps prefixed with a hash symbol (#). All input files will be channelled into the final process, prefixed with a greater-than symbol (>). List of modules excludes Core Cluster Flow modules. List valid at time of writing for Cluster Flow v0.4.
