# Supplementary material for: Cluster Flow: A user-friendly bioinformatics workflow tool
Source: F1000Res. 2017 May 2;5:2824. Originally published 2016 Dec 6. [Version 2] doi: 10.12688/f1000research.10335.2 (PMC5310375; doi:10.12688/f1000research.10335.2)
Supplement: Supplementary file 1 [file f1000research-5-12430-s0000.tgz › daa5df98-8df2-4025-8790-fd2eeda07a7b.pdf]

Cluster Flow: A user friendly bioinformatics workflow tool

Supplementary Information

Figure S1: Example Pipeline Script

```
/*
-----------------------------------
FastQ to Bismark Pipeline (RRBS Data)
-----------------------------------
This pipeline takes FastQ files as input, runs FastQC, Trim Galore,
then processed with Bismark (alignment, deduplication, methylation
extractor then tidying up and report generation). It requires a
genome index with a corresponding genome folder and bismark
converted genome.

Trim Galore is run with the RRBS parameter.
*/

#fastqc
#trim_galore    RRBS
    #bismark_align
        #bismark_methXtract
            #bismark_report
                >bismark_summary_report
```

Typical pipeline script showing analysis pipeline for *reduced representation bisulfite sequencing* (RRBS) data, from FastQ files to methylation calls with a project summary report. Pipeline steps will run in parallel for each read group for steps prefixed with a hash symbol (`#`). All input files will be channelled into the final process, prefixed with a greater-than symbol (`>`).

Table S1: List of supported tools

Module Name	Program Name	Description	Program Website
`bedToNrf`	-	Custom script - takes a BED file and computes the NRF (non-redundant fraction).	https://github.com/mel-astar/mel-ngs/blob/master/mel-chipseq/chipseq-metrics/CalBedNrf.pl
`bedtools_bamToBed`	bedtools	Convert BAM files to BED files and sort.	http://bedtools.readthedocs.io
`bedtools_intersectNeg`	bedtools	Filter out blacklisted regions from a BAM using a BED file.	http://bedtools.readthedocs.io
`bismark_align`	Bismark	Align Bisulfite data	http://www.bioinformatics.babraham.ac.uk/projects/bismark
`bismark_deduplicate`	Bismark	Remove duplicates from aligned Bisulfite data	http://www.bioinformatics.babraham.ac.uk/projects/bismark
`bismark_methXtract`	Bismark	Extract methylation calls from aligned Bisulfite data	http://www.bioinformatics.babraham.ac.uk/projects/bismark
`bismark_report`	Bismark	Create a sample report of Bismark analysis	http://www.bioinformatics.babraham.ac.uk/projects/bismark
`bismark_summary_report`	Bismark	Create a summary report of a Bismark project	http://www.bioinformatics.babraham.ac.uk/projects/bismark
`bowtie`	Bowtie 1 / 2	Run either Bowtie 1 or 2, depending on read length	
`bowtie1`	Bowite 1	Align reads using Bowtie 1	http://bowtie-bio.sourceforge.net
`bowtie2`	Bowtie 2	Align reads ugins Bowtie 2	http://bowtie-bio.sourceforge.net/bowtie2
`bwa`	BWA	Align reads using BWA	http://bio-bwa.sourceforge.net
`cf_download`	Cluster Flow	Download data from the web	http://clusterflow.io
`cf_merge_files`	Cluster Flow	Merge files using a regex (BAM, gzipped or text).	http://clusterflow.io
`deeptools_bamCoverage`	deepTools	Creates a bigWig coverage from from a BAM file	http://deeptools.readthedocs.io
`deeptools_bamFingerprint`	deepTools	Creates a fingerprint plot for ChIP-seq data	http://deeptools.readthedocs.io
`fastq_screen`	FastQ Screen	Runs FastQ Screen to check for contaminants	http://www.bioinformatics.babraham.ac.uk/projects/fastq_screen
`fastqc`	FastQC	Runs FastQC for basic QC metrics	http://www.bioinformatics.babraham.ac.uk/projects/fastqc
`featureCounts`	Subread featureCounts	Counts reads overlapping exons in a GTF file, grouped by gene.	http://bioinf.wehi.edu.au/featureCounts
`hicup`	HiCUP	Mapping and quality control for Hi-C data.	http://www.bioinformatics.babraham.ac.uk/projects/hicup

Module Name	Program Name	Description	Program Website
`hisat2`	HISAT2	Align RNA-seq reads with HISAT2	https://ccb.jhu.edu/software/hisat2
`htseq_counts`	HTSeq-count	Counts reads overlapping exons in a GTF file.	http://www-huber.embl.de/users/anders/HTSeq/doc/count.html
`kallisto`	Kallisto	Quantify abundances of transcripts from RNA-Seq data	https://pachterlab.github.io/kallisto
`multiqc`	MultiQC	Aggregates results from bioinformatics analyses across many samples into a single report	http://multiqc.info
`phantompeaktools_runSpp`	Phantom Peak Qual Tools	Runs cross correlation analysis on BAM files	https://github.com/kundajelab/phantompeakqualtools
`picard_dedup`	Picard	Mark duplicates in a BAM files	http://broadinstitute.github.io/picard
`preseq_calc`	Preseq	Calculate the complexity of a sequencing library	http://smithlabresearch.org/software/preseq
`rseqc_geneBody_coverage`	RSeQC	Calculate the average coverage across genes	http://rseqc.sourceforge.net
`rseqc_inner_distance`	RSeQC	Calculate the distance between reads for an RNA-seq library	http://rseqc.sourceforge.net
`rseqc_junctions`	RSeQC	QC plots for exon junction annoation and saturation	http://rseqc.sourceforge.net
`rseqc_read_GC`	RSeQC	Calculates a histogram showing the GC content of reads	http://rseqc.sourceforge.net
`samtools_bam2sam`	Samtools	Convert a BAM file to SAM	http://www.htslib.org
`samtools_dedup`	Samtools	Mark and remove duplicated sequences from BAM files	http://www.htslib.org
`samtools_sort_index`	Samtools	Sort and index a BAM file	http://www.htslib.org
`sra_abidump`	SRA-Tools	Extract csqual and csfasta files from .sra input	http://ncbi.github.io/sra-tools
`sra_fqdump`	SRA-Tools	Extract FastQ files from .sra input	http://ncbi.github.io/sra-tools
`star`	STAR	Align RNA data with STAR	https://github.com/alexdobin/STAR
`tophat`	TopHat	Align RNA data with TopHat	https://ccb.jhu.edu/software/tophat
`trim_galore`	TrimGalore!	Trim adapter sequence and low quality bases from reads	http://www.bioinformatics.babraham.ac.uk/projects/trim_galore

List of modules with tool description and URL. Core Cluster Flow modules excluded. List valid at time of writing for Cluster Flow v0.4.
