# Supplementary material for: Cluster Flow: A user-friendly bioinformatics workflow tool
Source: F1000Res. 2017 May 2;5:2824. Originally published 2016 Dec 6. [Version 2] doi: 10.12688/f1000research.10335.2 (PMC5310375; doi:10.12688/f1000research.10335.2)
Supplement: Peer review history

# Peer review: Referee response for version 2

**DOI:** 10.5256/f1000research.12430.r22445

**Published:** 2017-05-03

**Authors:** Alastair R. W. Kerr

**Affiliations:** 1 Wellcome Trust Centre for Cell Biology, University of Edinburgh, Edinburgh, UK

I thank the authors for thoroughly addressing the points raised. I have no further suggestions for the manuscript.

I have read this submission. I believe that I have an appropriate level of expertise to confirm that it is of an acceptable scientific standard.

# Peer review: Referee response for version 1

**DOI:** 10.5256/f1000research.11134.r18293

**Published:** 2017-02-16

**Authors:** David R. Powell

**Affiliations:** 1 Monash Bioinformatics Platform, Monash University, Clayton, VIC, Australia

This paper describes a pipeline tool, Cluster Flow,  (http://clusterflow.io/) specifically for bioinformatics processing.  Cluster Flow is well documented, and comes with many pipelines, and modules.  Pipelines are built by combining modules.  Modules define how to run specific tools, including the CPU and RAM requirements.  The tool works by specifying a pipeline to run, which then creates a shell script that either submits jobs to a cluster or runs locally depending on configuration.

 Cluster Flow is designed to be simple to use, but it does lack basic pipeline features such as being able to automatically re-run stages of a pipeline.

 It is not clear whether parameters can be changed when running a pipeline.  For example, selecting different adaptors for trimming, or different mapping thresholds for a short read aligner.  While Cluster Flow is designed to be simple, it seems such a feature would be commonly needed.

I have read this submission. I believe that I have an appropriate level of expertise to confirm that it is of an acceptable scientific standard.

# Response

**Published:** 2017-04-25

**Authors:** Philip Ewels

**Affiliations:** Stockholm University, Sweden

We thank the reviewer for this review. Pipeline parameters can indeed be changed (for example, adapters and trimming lengths) using the --params command line option. New documentation about this has been written and mention of it added to the manuscript (section: *Modules and pipelines*).

# Peer review: Referee response for version 1

**DOI:** 10.5256/f1000research.11134.r18292

**Published:** 2017-02-13

**Authors:** Stephen Taylor, Jelena Telenius

**Affiliations:** 1 Computational Biology Research Group (CBRG), Weatherall Institute of Molecular Medicine (WIMM), John Radcliffe Hospital, University of Oxford, Oxford, UK; 2 Weatherall Insitutute of Molecular Medicine (WIMM), University of Oxford, John Radcliffe Hospital, Headington, Oxford, UK

The authors present a useful automation pipeline for institutes, where significant amounts of similar analyses are run on daily basis.  It’s nice that potential users can get an idea of software by using the interactive web based terminal session (on http://clusterflow.io/). We recommend the software should be published but we have the following comments/questions.

 1) We installed the software fairly easily to run in ‘local mode’, although we couldn’t get it to run using Sun Grid Engine. It would be useful to put more documentation and/or examples here.

 2) How easy it is to add non Perl code to the software?

 It appears Perl is the main language to configure the pipelines but we were wondering about other languages. Are there standard procedures or templates for including R scripts and passing parameters to them, for example?

 3) Can one fine-tune the pipeline while running it?

 In contrast to adding changes to the pipelines, or adding new tools to the pipelines ( which is more a system admin / senior bioinformatician task ), one often needs to make frequent calls about "which parameters suit the analysis of this sequencing library the best" e.g. Thresholds for peak calling in ChIP-Seq. Can such thresholds be easily applied running the pipeline on the fly?

 4) Which kind of visualisation/report generating software do the authors recommend?

 As the pipeline produces a folder full of output results, it makes sense to have software to inspect these results. Which kind of software do you recommend to be used to this kind of task? Is there a concept of building reports? For example, is it recommended to use Labrador with CF (https://github.com/ewels/labrador)?

 5) How do the authors envisage managing multiple versions of very similar pipelines across different users and use cases without things becoming confusing and to encourage reuse of pipelines, rather than just creating new instances?

We have read this submission. We believe that we have an appropriate level of expertise to confirm that it is of an acceptable scientific standard.

# Response

**Published:** 2017-04-25

**Authors:** Philip Ewels

**Affiliations:** Stockholm University, Sweden

Many thanks for your time in reading the Cluster Flow manuscript and your helpful comments. We have revised the manuscript to address these points and are grateful for the help in improving the quality of the paper. Responses to specific comments are described below:

***1) We installed the software fairly easily to run in ‘local mode’, although we couldn’t get it to run using Sun Grid Engine. It would be useful to put more documentation and/or examples here.***

 We have extended the installation documentation available on the website. A walkthrough screencast tutorial is available on the Cluster Flow homepage (and YouTube). More concrete examples are difficult due to the varying setups of different clusters, though we continue to provide support via GitHub issues and e-mail.

***It appears Perl is the main language to configure the pipelines but we were wondering about other languages. Are there standard procedures or templates for including R scripts and passing parameters to them, for example?***

 Any language can be used for software modules, however Perl is recommended because of the available Cluster Flow functions which greatly simplify the interaction with the core program. There have been example modules bundled with Cluster Flow for Python and R, but they are difficult to maintain with the changes to the core Cluster Flow code and not advertised as a result. From experience, we find that it is usually easier to write a simple perl module is written which in turn executes downstream custom scripts.

***In contrast to adding changes to the pipelines, or adding new tools to the pipelines ( which is more a system admin / senior bioinformatician task ), one often needs to make frequent calls about "which parameters suit the analysis of this sequencing library the best" e.g. Thresholds for peak calling in ChIP-Seq. Can such thresholds be easily applied running the pipeline on the fly?***

 Whilst parameters and thresholds cannot be altered once a pipeline is running, it is possible to tweak such settings when launching an analysis. This is done by using the --params command line flag (can also be specified within pipeline files). We were somewhat shocked to realise that there was no documentation of this feature anywhere and have added a new section to the Cluster Flow documentation. This describes all available --params for every module. We have added mention of this to the manuscript (section: *Modules and pipelines*).

***As the pipeline produces a folder full of output results, it makes sense to have software to inspect these results. Which kind of software do you recommend to be used to this kind of task? Is there a concept of building reports? For example, is it recommended to use Labrador with CF (https://github.com/ewels/labrador)?***

 Labrador is able to view some results from Cluster Flow pipelines (such as the .html completion report). However, the authors have written another tool called MultiQC which is able to summarise all results from a pipeline into a single html file [1]. See http://multiqc.info for more information.

***5) How do the authors envisage managing multiple versions of very similar pipelines across different users and use cases without things becoming confusing and to encourage reuse of pipelines, rather than just creating new instances?***

 This is of some concern to the authors, and we encourage users to submit new modules and pipelines back to the main Cluster Flow repository so that they are available to everyone. However, we do not want to sacrifice flexibility, and so aim for maximum traceability by saving pipeline and module information for every run. A central repository for modules and pipelines has also been discussed (see comments to review 1 above) but is not yet being actively worked on.

 [1] MultiQC: Summarize analysis results for multiple tools and samples in a single report. Philip Ewels, Måns Magnusson, Sverker Lundin and Max Käller. *Bioinformatics* (2016) doi: 10.1093/bioinformatics/btw354

# Peer review: Referee response for version 1

**DOI:** 10.5256/f1000research.11134.r18295

**Published:** 2016-12-19

**Authors:** Alastair R. W. Kerr, Shaun Webb

**Affiliations:** 1 Wellcome Trust Centre for Cell Biology, University of Edinburgh, Edinburgh, UK

As the software is available and in use in multiple institutions (and thus tried and tested), I have no problems with accepting the manuscript. I feel that the manuscript and/or the linked documentation would benefit from some changes noted below.

**Install**

 Having a copy of the install instruction in the downloaded tarball would be useful.

 The “cf” executable uses the FindBin Perl module to establish the location of the script and hence the relative path to the CF Perl modules. Therefore the install must add the clusterflow directory to the PATH and would not function if the “cf” executable was symlinked to a directory on the PATH. This should be made clear in the install instructions although this is alluded to in the manuscript.

**Adding genomes**

 The program can add genomes from installed locations in the filesystem. A helper script to autoinstall from Ensembl/UCSC public sites would be a benefit. Moreover it is unclear if missing index files for mapping programs are generated automatically and permanently stored when running pipelines. This would be useful and easy to implement.

**Metadata**

 I am glad to see the workflow captures metadata such as software versions and this should be highlighted in the manuscript. A reporting tool to extract this information, perhaps in a tabular format, from the log files would be useful.

**Reproducibility**

 Output from the pipelines are depended on the software versions on the PATH. This is not ideal and an easy way to configure software versions would be useful to allow reproducible pipelines. I assume that “modules” are what the maintainers imagine most people would use? Docker would have been a nice solution.

**Adding programs**

 There is information in the on-line documentation to add new programs to clusterflow by writing wrappers. This functionality should be noted in the manuscript.

**Upgrades**

 It is unclear how clusterflow can upgraded (I assume that new tarball needs to be downloaded) and whether there are repositories for new pipelines or tools. For example it would be useful for a community facility for depositing new tools and pipelines.

**Language**

 Is providing compatibility with the common workflow language [CWL] ^1^​​​​​​​ a possibility or a likelihood?

**Resources**

 I would like more detail on the following:

 How exactly is runs/threads/memory managed on a single node cluster? How happens if multiple users each run cf? Are instances aware of each other? Do the scripts check how many jobs are running or how much free memory is available?

We have read this submission. We believe that we have an appropriate level of expertise to confirm that it is of an acceptable scientific standard.

## References

[1] AmstutzPTijanićNChapmanBChiltonJHeuerMKartashovALeehrDMénagerHNedeljkovichMScalesMSoiland-ReyesSStojanovicL: Common Workflow Language, v1.0. *Figshare*.2016;

# Response

**Published:** 2017-04-25

**Authors:** Philip Ewels

**Affiliations:** Stockholm University, Sweden

Many thanks for your time in reading the Cluster Flow manuscript and your helpful comments. We have revised the manuscript to address these points and are grateful for the help in improving the quality of the paper. Responses to specific comments are described below:

***Having a copy of the install instruction in the downloaded tarball would be useful.***

 All of the Cluster Flow documentation is included with the downloaded tarball as markdown files within the ‘docs’ directory. However, we agree that this could be more visible. We have rewritten the main README.md file (also shown on the GitHub front page) to include brief installation instructions with a link to the longer documentation.

***The “cf” executable uses the FindBin Perl module to establish the location of the script and hence the relative path to the CF Perl modules. Therefore the install must add the clusterflow directory to the PATH and would not function if the “cf” executable was symlinked to a directory on the PATH. This should be made clear in the install instructions although this is alluded to in the manuscript.***

 Thank you for alerting us to this issue. We have updated Cluster Flow to use $RealBin instead of $Bin, which makes it work with symlinks.

***The program can add genomes from installed locations in the filesystem. A helper script to autoinstall from Ensembl/UCSC public sites would be a benefit. Moreover it is unclear if missing index files for mapping programs are generated automatically and permanently stored when running pipelines. This would be useful and easy to implement.***

 We agree that such a helper script would be useful and will look into writing this for a future release. At the time of writing, missing index files are not automatically generated. We recommend using illumina iGenomes where appropriate. We have written a helper tool to add centralised reference genomes for users running Cluster Flow on the Swedish UPPMAX clusters.

***I am glad to see the workflow captures metadata such as software versions and this should be highlighted in the manuscript. A reporting tool to extract this information, perhaps in a tabular format, from the log files would be useful.***

 Mention of this has been added to the manuscript (section: *Notifications and logging*). In the new Cluster Flow release (v0.5), modules have been updated to extract and standardise the version numbers. These are now included in summary e-mails and parsed by a new Cluster Flow module written for MultiQC. MultiQC produces also machine-readable versions of this data (tsv, csv, json or yaml).

***Output from the pipelines are depended on the software versions on the PATH. This is not ideal and an easy way to configure software versions would be useful to allow reproducible pipelines. I assume that “modules” are what the maintainers imagine most people would use? Docker would have been a nice solution.***

 As the reviewer suggests, Cluster Flow was primarily designed for use with environment modules as a method to standardise tools used and support for that is built in. We have added to the Cluster Flow submission log file, which now contains information about all loaded environment modules, all directories currently on the PATH, the current user and information about the compute environment. Users are able to run Cluster Flow inside a docker container if desired, using local mode.

***There is information in the on-line documentation to add new programs to clusterflow by writing wrappers. This functionality should be noted in the manuscript.***

 This is now described within the manuscript (section: *Modules and pipelines*).

***It is unclear how clusterflow can upgraded (I assume that new tarball needs to be downloaded) and whether there are repositories for new pipelines or tools. For example it would be useful for a community facility for depositing new tools and pipelines.***

 This is correct, Cluster Flow is updated by replacing the program files with a new tarball download. A community repository for Cluster Flow modules and pipelines has previously been discussed, and we hope to be able to work on this project in the future.

***Is providing compatibility with the common workflow language [CWL]​​​​​ a possibility or a likelihood?***

 The authors have looked into such compatibility in the past, including discussing the point with Michael Crusoe when he visited our institute to present CWL. Unfortunately, due to differing assumptions and architectures within the two systems it seems unlikely that this will be pursued.

***How exactly is runs/threads/memory managed on a single node cluster? How happens if multiple users each run cf? Are instances aware of each other? Do the scripts check how many jobs are running or how much free memory is available?***

 Running Cluster Flow in local mode on a single node cluster is fairly simplistic. There is no resource management and instances are not aware of each other. It was primarily written for easy testing and low-throughput runs where this will not be a problem. If running a lot of jobs on a single server we recommend installing a job management system such as SLURM. We have added a sentence describing this to the manuscript (section: *Use Cases*).
